# New Splice Site Acceptor Mutation in *AIRE* Gene in Autoimmune Polyendocrine Syndrome Type 1

**DOI:** 10.1371/journal.pone.0101616

**Published:** 2014-07-02

**Authors:** Mireia Mora, Felicia A. Hanzu, Marta Pradas-Juni, Gloria B. Aranda, Irene Halperin, Manuel Puig-Domingo, Sira Aguiló, Eduardo Fernández-Rebollo

**Affiliations:** 1 Department of Endocrinology and Nutrition, Hospital Clinic, Barcelona, Spain; 2 Laboratory of Endocrine Disorders, Institut d’Investigacions Biomèdiques August Pi I Sunyer (IDIBAPS), Barcelona, Spain; 3 Spanish Biomedical Research Centre in Diabetes and Associated Metabolic Disorders (CIBERDEM), Barcelona, Spain; 4 Diabetes and Obesity Research Laboratory - Institut d’Investigacions Biomèdiques August Pi i Sunyer (IDIBAPS), Barcelona, Spain; 5 Department of Endocrinology and Nutrition, Hospital Germans Trias i Pujol Research Institut and Hospital, Universitat Autònoma de Barcelona, Badalona, Spain; 6 Department of Internal Medicine, Hospital Clínic, Barcelona, Spain; University of Valencia, Spain

## Abstract

Autoimmune polyglandular syndrome type 1 (APS-1, OMIM 240300) is a rare autosomal recessive disorder, characterized by the presence of at least two of three major diseases: hypoparathyroidism, Addison’s disease, and chronic mucocutaneous candidiasis. We aim to identify the molecular defects and investigate the clinical and mutational characteristics in an index case and other members of a consanguineous family. We identified a novel homozygous mutation in the splice site acceptor (SSA) of intron 5 (c.653-1G>A) in two siblings with different clinical outcomes of APS-1. Coding DNA sequencing revealed that this *AIRE* mutation potentially compromised the recognition of the constitutive SSA of intron 5, splicing upstream onto a nearby cryptic SSA in intron 5. Surprisingly, the use of an alternative SSA entails the uncovering of a cryptic donor splice site in exon 5. This new transcript generates a truncated protein (p.A214fs67X) containing the first 213 amino acids and followed by 68 aberrant amino acids. The mutation affects the proper splicing, not only at the acceptor but also at the donor splice site, highlighting the complexity of recognizing suitable splicing sites and the importance of sequencing the intron-exon junctions for a more precise molecular diagnosis and correct genetic counseling. As both siblings were carrying the same mutation but exhibited a different APS-1 onset, and one of the brothers was not clinically diagnosed, our finding highlights the possibility to suspect mutations in the *AIRE* gene in cases of childhood chronic candidiasis and/or hypoparathyroidism otherwise unexplained, especially when the phenotype is associated with other autoimmune diseases.

## Introduction

Autoimmune polyendocrine syndrome type 1 (APS-1, OMIM 240300), previously known as Autoimmune polyendocrinopathy-candidiasis-ectodermal dystrophy (APECED), is a childhood-onset monogenic autoimmune disease [1], [2]. APS-1 is highly variable clinically, genetically, and serologically, and its traditional clinical diagnosis requires the presence of at least two of three hallmark conditions: chronic mucocutaneous candidiasis, hypoparathyroidism and Addison’s disease [2]. However, patients with APS-1 also may exhibit additional autoimmune diseases, including type 1 diabetes mellitus (T1D), hypothyroidism, vitiligo, alopecia, autoimmune hepatitis, pernicious anemia, and asplenism [3]. The range of these secondary autoimmune disorders is broad and variable [4].

**Figure 1:**
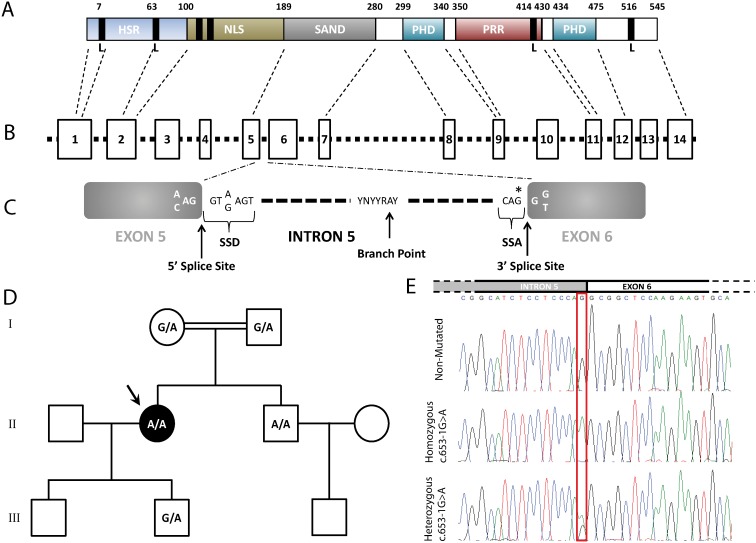
AIRE overview family pedigree and detection of the c.653-1G>A AIRE mutation. (A) Schematic AIRE protein representation showing the different protein domains: HSR domain (HSR), conserved bipartite nuclear localization signal (NLS), PHD zinc finger motif (PHD), proline-rich region (PRR), LXXLL motif (L), and SAND domain (SAND). (B) Schematic *AIRE* gene representation, where rectangles indicate exons and the dashed line the introns. Finally, (C) schematic representation of the consensus sequences for the 5′ splice site donor (SSD), branch site and 3′ splice site acceptor (SSA). The star indicates the mutation. (D) Pedigree of the Spanish consanguineous family. Genotypes were shown as wild-type (G/G), heterozygous (G/A) and homozygous (A/A) of the c.653-1G>A *AIRE* mutation. The arrow indicates the index case. (E) Above, the schematic representation of the junction between intron 5 and exon 6, and below, the direct sequence analysis of the *AIRE* gene identified homozygous carriers of the c.653-1G>A mutation in APS-1 patients, while heterozygous carriers were found in unaffected relatives (I-1, I-2, III-2) of the family.

Mutations in the autoimmune regulator (*AIRE*) gene are responsible for this rare autosomal, recessively inherited disease [5], [6]. The *AIRE* gene maps to 21q22.3 and consists of 14 exons spanning approximately 13 kb of genomic DNA, which encodes a protein made up of 545 amino acids (Figure 1A and 1B). The AIRE protein contains motifs indicative of its transcription factor function, including a conserved bipartite nuclear localization signal (NLS), two Plant Homedomain (PHD) zinc-finger motifs, four LXXLL nuclear receptor binding motifs (where L is leucine and X is any amino acid), and a proline rich region (PRR) [6], [7]. A SAND domain is common to AIRE and some homologous proteins and has been suggested to be a DNA binding domain [8]. The 100 NH2-terminal amino acids of AIRE form a homogenously staining region (HSR) domain also present in the Sp100 and Sp140 proteins, where it has been shown to be responsible for homodimerization and is required for their activation [7], [9]–[11] (Figure 1B). Mutations in this transcription factor determine the development of multi-organ autoimmune disease, as the activity of this autoimmune regulator governs the expression of diverse self-antigens in the thymus, preventing the maturation of auto-reactive T cells that escape thymic negative selection [12]–[14].

To date, more than a hundred different mutations of the *AIRE* gene have been identified in APS-1 patients, but only two major mutations (p.R257X and p.L323SfsX51) are responsible for 95% of the mutant alleles in APS-1 patients [15]–[17]. However, it is noteworthy that few *AIRE* mutations have been thoroughly studied [18] and on the other hand, with only one previous publication about an *AIRE* mutation reported in Spain [19].

Here we report the identification of a novel splice site acceptor (SSA) mutation in intron 5 of the *AIRE* gene (c.653-1G>A) in two siblings from a Spanish consanguineous family, with a different clinical outcomes in APS-1. Moreover, *in silico* and mRNA studies showed that this mutation results in a new SSA in intron 5, which uncovers a cryptic donor splice site in exon 5, generating a truncated protein with a premature termination codon (p.A214fs67X) downstream of exon 6, losing part of the SAND domain and the PHD1, PHD2 and PRR domains completely. Finally, we explored the Nonsense-mediated decay (NMD), a mechanism that eliminates mRNAs containing premature termination codons and limits the synthesis of abnormal proteins, which helps explain if NMD accounts for the different clinical outcomes between both homozygous carriers.

## Patients and Methods

### Patients

The index case affected by APS-1 and her unaffected available relatives were investigated (Figure 1D). The study was approved by the Institutional Review Board (Ethics Committee of Hospital Clinic of Barcelona, Spain) and has been conducted in accordance with the guidelines established in the Declaration of Helsinki, requiring a written informed consent by all participants before the study. All participants provided written informed consent to have the details of their cases published.

### Clinical data

The index case, a 52-yr-old woman (55.3 kg and 155 cm; II.1, figure 1D), was the first of two healthy children born to first cousin Spanish parents, without any previous family history for autoimmune diseases. The patient presents chronic mucocutaneous candidiasis, primary hypoparathyroidism, hypergonadotropic hypogonadism and intestinal dysfunction. Since childhood, she presented mucocutaneous and nail candidiasis, and she developed vaginal candidiasis during adulthood, as well as a chronic intestinal dysfunction. She was diagnosed with primary hypoparathyroidism at 42 years old based on paresthesias, muscle cramps and tetany, and an ionized calcium determination of 0.55 nM/l (normal range 1–1.2 nM/l) together with a parathormone (PTH) value of 10.1 pg/ml (normal range 10–65 pg/ml). During follow-up, chronic gastritis has been diagnosed with low levels of B12 vitamin and positive parietal cell antibodies. Currently, she requires 3 g of calcium carbonate, 0.75 µg of calcitriol and 1.2 g of magnesium lactate daily. She has presented several episodes of gastrointestinal dysregulation that have required modifications in the dosage of the aforementioned compounds. Recent analyses were positive for Anti-thyroid-peroxidase antibodies (TPOAb) positive (34 UI/ml in 2005 and 140 UI/ml in 2013), as well as for Anti-Glutamic acid decarboxylase antibodies (GADAb) (160 U/ml in 2013).

For his part, the brother of the index case (74.9 kg and 177 cm; II.2, figure 1D) is a 46-yr-old man with mucocutaneous and nail candidiasis since childhood and universal alopecia since his 40s. He also presents pyloric stenosis since birth, as well as hypospadia and chronic C hepatitis. Recent analyses were still negative for AcTPO antibodies, but were slightly positive for GADAb (1 U/ml in 2013).

I.1 and I.2 are first cousins, and their sons (cases II.1 and II.2) were born in Barcelona, as well as their respective sons, III.1 and III.2 from II.1, and III.3 from II.2. Cases III.1, III.2 and III.3 are healthy men of 26, 22 and 12 years old, respectively. Case I.1 is a 78-yr-old woman with type 2 diabetes and hypertension, having undergone a hysterectomy and surgical intervention for inguinal hernia. Case I.2 is an 84-yr-old man with dyslipidemia, hypertension, and ischemic cardiopathy and carries a pacemaker. Case III.2 is a healthy young man, without any clinical features suggesting APS-1. Available clinical characteristics and analytical data for all individuals are reported in table 1.

**Table 1:** Clinical features and analytical data.

	I.1	I.2	II.1	II.2	III.2
Sex	F	M	F	M	M
**Age at onset (years)**	-	-	42	-	-
**Age at last exam (years)**	78	84	52	47	22
**Candidiasis**	-	-	+	+	-
**Hypoparathyroidism**	-	-	+	-	-
**Addison’s disease**	-	-	-	-	-
**Hypogonadism**	-	-	+	-	-
**IDDM**	-	-	-	-	-
**T2D**	+	-	-	-	-
**Hypothyrodism**	-	-	-	-	-
**Atrophic gastritis**	-	-	+	-	-
**Pernicious anemia**	-	-	+	-	-
**Autoimmune hepatitis**	-	-	-	-	-
**Malabsorption**	-	-	+	-	-
**Keratitis**	-	-	-	-	-
**Alopecia**	-	-	-	+	-
**Vitiligo**	-	-	-	-	-
**Enamel hypoplasia**	-	-	+	+	-
***AIRE*** ** genotype**	G/A	G/A	A/A	A/A	G/A
**Calcium (8.5–10.5 mg/dl)**	8.8	9	4.5	10	10
**Phosphorus (2.3–4.3 mg/dl)**	-	3.5	4.7	2.9	3.7
**Magnesium (1.8–2.6 mg/dl)**	-	2.1	1.7	2.2	2.1
**PTH (10–65** **pg/ml)**	27	99	<2	16	40
**25-hidroxi-vitaminD (30–100 ng/ml)**	23.6	14.1	40.7	28	26.8
**ACTH (10–60** **pg/ml)**	28	35	36	34	24
**Basal cortisol (10–25** **mcg/dl)**	17.3	13.1	24	20	16.4
**Anti 21-hidroxilase Ab (<1 U/ml)**	<0.5	<0.5	0.7	<0.5	0.5
**Anti-peroxidase Ab (<35** **UI/ml)**	<28	<28	32	44	32
**Anti-tiroglobulin Ab (<60** **UI/ml)**	<15	<15	140	<15	<15
**Parietal cell Antibodies**	-	-	+++, 80 URF	-	-
**B12 vitamine (250–1050** **pg/ml)**	-	-	129	424	-
**Folic acid (250–1050 ng/ml)**	-	-	438.9	284	-
**Insulin growth factor (171–333 ng/ml)**	-	-	62	87	-
**Luteotropin (LH) (1.5–7.5** **U/l)**	-	-	49.97	1.95	-
**Foliculoestimuline (FSH) (1.7–8** **U/l)**	-	-	113.54	2.4	-
**Anti-GAD (<0.5 U/ml)**	-	-	160	1	-
**Anti-IA-2Ab (<0.75 U/ml)**	-	-	0.3	0.2	-

Abbreviations: IDDM (Insulin-dependent Diabetes Mellitus), T2D (Type 2 Diabetes), PTH (Parathyroid hormone), ACTH (Adrenocorticotropic hormone), GAD (Glutamic acid decarboxylase), IA-2 (Islet Antigen 2) and Ab (antibodies).

### Antibody, hormonal and biochemical determinations

Main biochemical, hormonal and antibody determinations after overnight fasting were measured in serum in the laboratories of the Hospital Clínic (Barcelona, http://cdb.hospitalclinic.org) using specific standard validated assays.

### Molecular studies

#### DNA and PCR

Genomic DNA was extracted from peripheral blood leucocytes using the QIAamp DNA Blood Midi Kit (Qiagen, Hilden, Germany). Fourteen exons and their flanking exon-intron boundaries of the *AIRE* gene were amplified by PCR, which was performed with PrimeSTAR HS DNA Polymerase (Takara Bio, Japan). The PCR products were purified using a QIAquick PCR Purification Kit (Qiagen) and sequenced by BigDye Terminator v3.1 Cycle Sequencing Kit (Applied Biosystems). Primer sequences are shown in Table S1.

### Real-time quantitative PCR

#### RNA isolation

Total RNA from blood samples was isolated using the QIAamp RNA Blood Mini Kit (Qiagen, Hilden, Germany) according to the manufacturer’s instructions.

#### cDNA synthesis

Reverse transcription reaction was performed using a commercially available set of High Capacity cDNA Reverse Transcription Kit (Applied Biosystems, USA). cDNA was prepared from 1 µg of mRNA, with random hexamer primers, according to the manufacturer’s instructions.

Quantitative Real-time PCR (qRT-PCR) was performed on an ABI Prism 7900HT Sequence Detection System (Applied Biosystems, Foster City, CA, USA). The reaction was performed using the SYBR Premix Ex Taq (Tli RNase H Plus; Takara-Clontech), and dissociation curve analysis of the PCR products was performed at the end of amplification to verify single product amplification.

The relative quantification of gene expression in each sample was analyzed using SDS Software version 2.4 (Applied Biosystems). Relative expression of each gene to Beta Actin (β-actin) was calculated using the accurate cycle threshold method [20]. The experiment was run twice in triplicate. Primer sequences are shown in Table S1.

### Mutational and *in Silico* Splicing Analyses

The mutation was identified by comparing with the reported reference sequence (NM_000383.2) and further analyzed in control individuals. Since the mutation could affect mRNA splicing, both wild-type sequence and altered sequence were analyzed *in* *silico* using the following splice site prediction web interfaces: SplicePort (SP: http://spliceport.cbcb.umd.edu/) [21], Human Splice Finder (HSF: http://www.umd.be/HSF/) [22] and Alternative Splice Site Predictor (ASSP: http://wangcomputing.com/assp/index.html) [23]. Predictions were made using default settings.

After carrying out the splice-site and cDNA sequence prediction *in silico*, we analyzed the putative protein sequence using Expasy software (http://web.expasy.org/translate/software), in order to predict whether this amino acid substitution affects protein function using the Protein Variation Effect Analyzer (Provean: http://provean.jcvi.org/index.php) [24].

### Protein Structure Prediction

3D protein structure of mutant and normal AIRE proteins was predicted using the I-TASSER fold recognition method [25]. The predicted mutant and normal structures were superimposed and aligned using PyMol software.

## Results

### Identification of a new *AIRE* gene mutation and analysis of the new generated splice site

**Figure 2:**
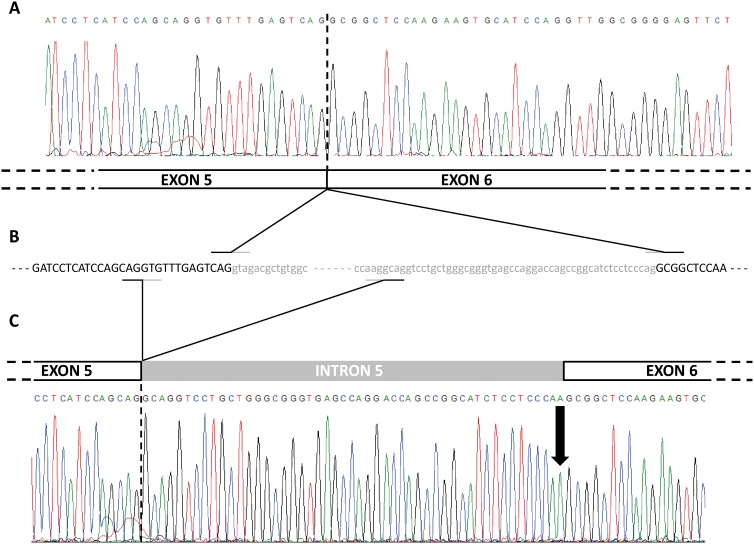
Sequencing representation of the cDNA analysis. (A) Chromatogram and schematic representation of the non-mutated exon 5 and 6 junction. (B) Partial gDNA sequence of exon 5, intron 5 and exon 6. Finally, (C) chromatogram and schematic representation of the mutated allele, including exon 5, part of intron 5 and exon 6. The arrow indicates the mutation.

Based on the clinical features of the index case, which supported the diagnosis of APS-1, mutational screening of the *AIRE* gene was carried out including the 14 coding exons and the intronic boundaries. Direct sequencing of the *AIRE* gene revealed that not only the presumed APS-1 index case, but also her brother (cases II.1 and II.2, respectively), were homozygote for splicing mutation c.653-1G>A at the last nucleotide of intron 5 (Figure 1E), which belongs to the splice site acceptor (SSA) conserved sequence [26] (Figure 1C). The mutation was also detected in heterozygous state in the other healthy relatives examined (I.1, I.2 and III.2; Figure 1D), but was not detected in 100 healthy control subjects. This mutation has not been previously reported in the scientific literature. Surprisingly, the use of new SSA in intron 5 entails the uncovering of a cryptic exonic splice site donor (SSD) at exon 5 generating an aberrant transcript, as shown by cDNA sequencing in both homozygous and heterozygous carriers (Figure 2). These results were consistent with *in silico* analysis by Human Splicing Finder, Splicing Port and Alternative Splice Site Predictor software (Table 2).

**Table 2:** Splice site prediction software output.

DESCRIPTION	ACCEPTOR/DONOR	EXON/INTRON	SPLICE SITE SEQUENCE	HSF		SP	ASSP	
				CONSENSUS VALUE	SCORE	SCORE	SCORE	CONFIDENCE
Constitutive	DONOR	EXON 5	CAGgtagac	77.32	6.89	0.48639	6.866	0.864
Cryptic	DONOR	EXON 5	CAGgtgtt	66.35	2.28	0.25713	7.430	0.512
Constitutive	ACCEPTOR	INTRON 5	cctcccagGCG	73.83	5.05	0.55301	7.595	0.822
Mutated	ACCEPTOR	INTRON 5	ctcctcccaaGCG	93.55	2.71	–0.43946	3.443	0.486
Cryptic	ACCEPTOR	INTRON 5	gccaagGCA	72.00	2.53	–2.14118	–1.914	0.941

The scores of the preprocessing models reflect the splice site strength. Abbreviations: HSF (Human Splice Finder), SP (Splice Port) and ASSP (Alternative Splice Site Predictor).

At the amino acidic level, this mutation at the SSA generates a truncated protein, and, along these lines, the Provean software predicts that this amino acid substitution has a deleterious impact (Score = −620; variants with score equal or below −2.5 are considered deleterious) on the biological function of the protein.

**Figure 3:**
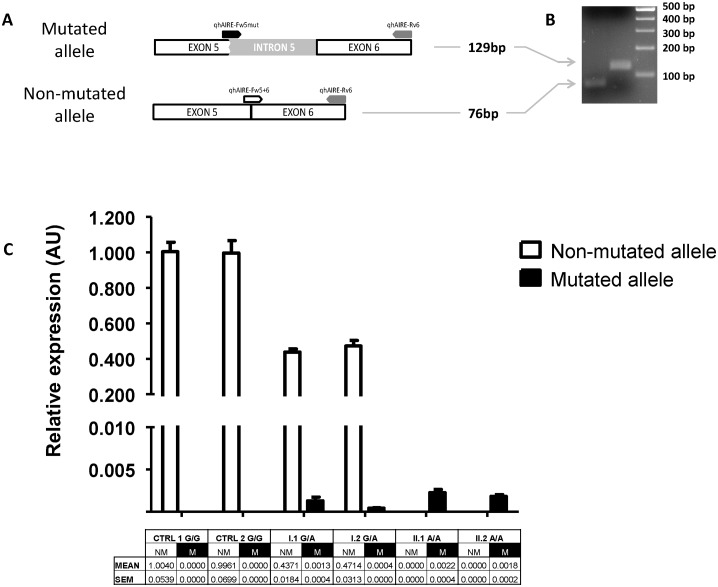
Nonsense pre-mRNA mediated decay analysis. (A) Schematic representation of the non-mutated and mutated allele analysis, with the specifically designed primers positions, including the non-mutated forward (white), the mutated forward (black) and the common reverse (grey). (B) Agarose gel electrophoresis of the qRT-PCR products revealed that the cDNA fragment of 76-bp corresponds to the predicted wild-type transcript, whereas the cDNA large fragment of 129-bp corresponds to the aberrant transcript. (C) Expression results of each allele normalized to that of the control G/G carriers for the individuals studied. The table below includes the mean relative expression and the standard error mean (SEM) for the non-mutated (NM) and mutated (M) alleles.

To assess if the generation of a premature termination codon could trigger the nonsense-mediated mRNA decay mechanism, we performed a qRT-PCR for the non-mutated (G/G) and mutated (A/A) alleles, from the index case, her brother, both parents and two healthy controls. We designed a forward primer specific for the non-mutated allele covering the junction between exon 5 and 6, another specific forward primer for the junction between the last nucleotides of the truncated exon 5 and first nucleotides from the intron 5, and finally a common reverse primer in exon 6 (Figure 3A and 3B). The relative expression of the non-mutated and mutated alleles was normalized to that of the healthy G/G control individuals. The results for the non-mutated allele showed normal expression levels for both controls, an absence of expression for the index case and her brother, and half expression levels for both heterozygous parents, as expected (Figure 3C). On the other hand, the expression results for both homozygous or the heterozygous carriers of the mutated allele were very low and even absent for the healthy controls, suggesting the activation of the NMD process in the degradation of the mRNA from the mutated allele. These results showed similar expression patterns for the mutant allele in both homozygous carriers, ruling out the possibility that the NMD could account for the differences in clinical features between siblings.

**Figure 4:**
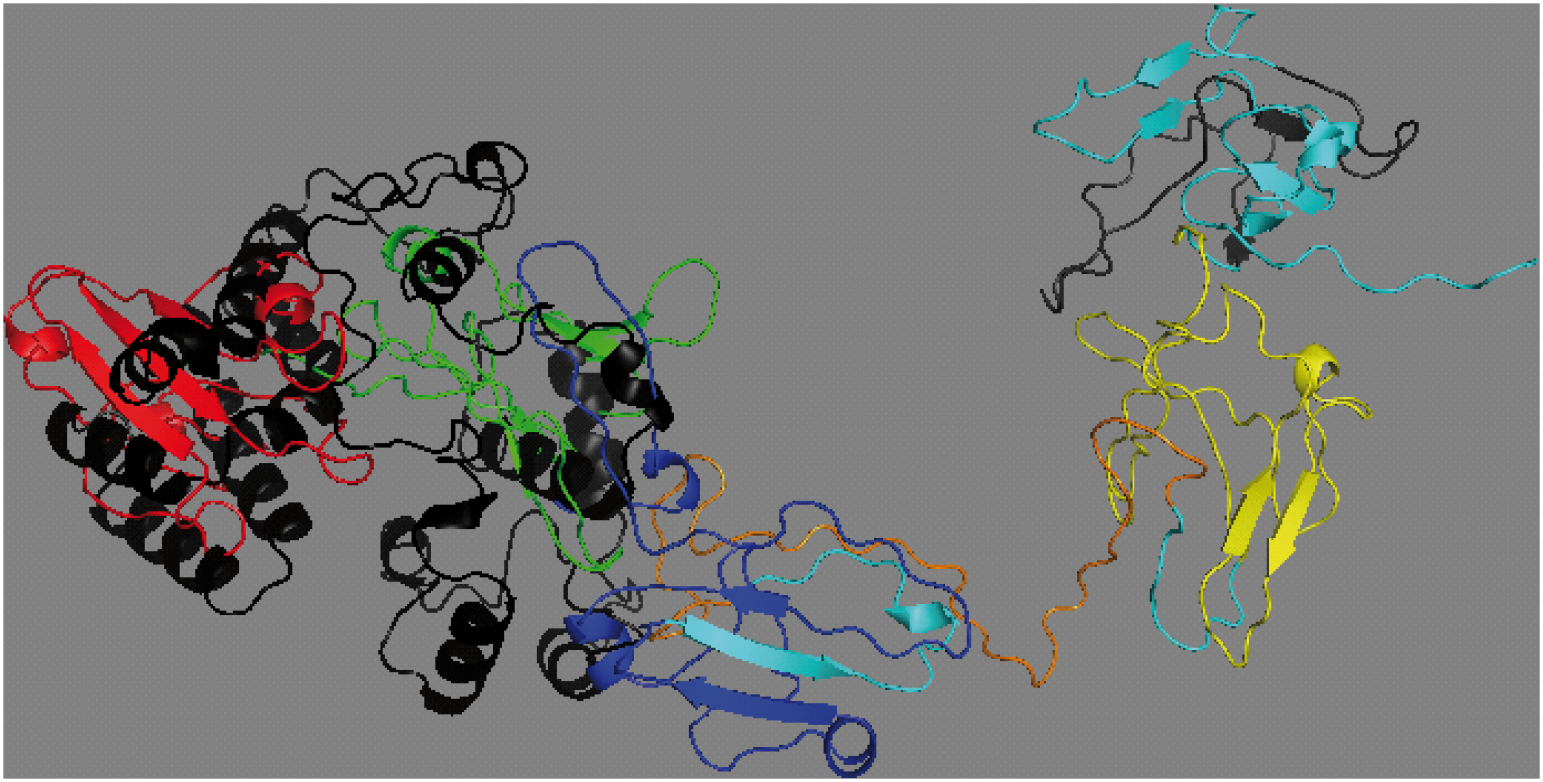
Tertiary schematic representation of the aligned non-mutated and mutated AIRE proteins. The non-mutated AIRE protein shows the different functional domains: HSR in red, NLS in green, SAND in blue, PHD in orange and PRR in yellow. The mutated AIRE protein, for its part, is represented in black.

Although previous *in silico* analyses have shown that this mutation encodes for a truncated protein, both proteins still share 213 amino acids, including the homodimerization and activation domain HSR, and part of the DNA binding domain SAND. We then decided to analyze the predicted protein tertiary structure based on I-TASSER, discovering that the mutated protein (score −4.9) presents a different tertiary structure (Figure 4).

### Discordant clinical characteristics of APS-1 patients in a consanguineous Spanish family

In this study we examined two siblings from a consanguineous Spanish family with autosomal recessive APS-1. They were born from healthy consanguineous parents (first cousins; Figure 1D).

The index case (II.1) was a middle-aged woman who was diagnosed with severe APS-1 symptoms including chronic mucocutaneous candidiasis, primary hypoparathyroidism, hypergonadotropic hypogonadism and intestinal dysfunction at 42 years of age. Recent analyses have tested positive for both GAD and anti-peroxidase antibodies, and she has been diagnosed with atrophic gastritis.

Although her brother (II.2) also carries the same homozygous mutation, he did not present hypoparathyroidism. However, he showed early signs of mucocutaneous candidiasis, and additional symptoms appeared at adulthood, including vitiligo and alopecia. Both homozygous carriers showed normal cortisol levels and did not test positive for antibodies for 21-hidroxilase, which initially rules out Addison’s disease.

Finally, although several heterozygous relatives of APS-1 patients have been reported to have various autoimmune diseases such as rheumatoid arthritis, without any of the APS-1 major syndromes [27], we did not find any APS-1 features in the relatives studied.

## Discussion

Since *AIRE* was first described as the APS-1 gene in 1997, more than a hundred different mutations of the *AIRE* gene have been identified in APS-1 patients, including two major mutations (p.R257X and p.L323SfsX51) which are responsible for 95% of the mutant alleles in APS-1 patients [15]–[17]. The rest of the reported mutations are also other missense or nonsense mutations, while splicing mutations are less common [28].

This autosomal recessive disorder is highly prevalent in certain genetically isolated populations, such as Finns, Sardinians and Iranian Jews [28], [29], but is rare in the Spanish population, as just one case with a 13 bp deletion in exon 8 has been previously described [19].

In the present study, we identified two Spanish siblings, brother and sister, who carried a new homozygous mutation of the *AIRE* gene in the SSA of intron 5. The c.653-1G>A mutation turns the trinucleotide sequence CAG into CAA, losing the consensus SSA but creating a new SSA upstream in the intron 5, as *in silico* analysis predicted and mRNA analysis demonstrated. But the c.653-1G>A mutation not only induces the use of a new SSA upstream of intron 5, but also produces the cryptic SSD activation inside the proper exon 5, as *in silico* analysis also predicted (Table 2).

This mutation at the SSA is predicted to generate a truncated protein, thus we theorized that the nonsense-mediated mRNA decay (NMD) is activated. NMD is a surveillance pathway, whose main function is to reduce errors in gene expression by abolishing mRNA transcripts that contain premature termination codons. This prevents the translation of aberrant transcripts and limits the synthesis of abnormal proteins [30], such as the transcripts generated from the mutated allele. Thus, NMD could take on a role as a modifier of the phenotypic and clinical consequences of the premature termination codon. This prompted us to analyze NMD in both homozygous and heterozygous carriers, studying the expression levels of the mutated and also non-mutated alleles. Our findings revealed a highly reduced expression of the mutated allele as an expected result of the activation of the NMD process (Figure 3B), but the reduced expression of the mutated allele was similar in both siblings. This suggests that the protective function of NMD contributes equally, ruling out the possibility that this mechanism was involved in the different clinical outcomes of APS-1 in these cases.

Although the expression of the mutated allele was very low and the mutation at the SSA sequence was expected to generate a truncated protein, the mutated and non-mutated proteins shared the first 213 amino acids, including the conserved bipartite nuclear localization signal domain (NLS) and the homogenously staining region (HSR) responsible for homodimerization. This made us wonder if the mutated protein could conserve in part the tertiary structure for these first domains, in which case perhaps it could exert partially its function. However, the alignment of the predicted tertiary structure for the mutated protein and the non-mutated AIRE protein (Figure 4) showed no-matching for the first domains, suggesting loss of functionality for the mutated protein.

Clinical picture of APS-1 is characterized by sequentially occurring diseases, with great variation in the severity and time course of the conditions among families and members of the family. Although candidiasis is not specific to APS-1, it is almost always present (>94% at 10 years of age and >97% at 30 years of age) [31], occurring in both homozygous members of this family. The high prevalence of candidiasis leads us to suspect APS-1, especially when clinical presentation takes place during childhood. The severity of its clinical manifestations varies widely and its most disabling feature may lead to the development of squamous cell carcinoma. However, both members of the family in question presented a less severe course, with chronic nail but intermittent oral candidiasis, since onset in childhood. Hypoparathyroidism is one of the first endocrine features of APS-1 [32] and is reported in 70–93% of the cases [33] but varies according to gender, affecting 98% of female patients, and only 71% of male patients [34]. There is no reasonable explanation for these gender differences, besides a clearly higher incidence of primary gonadal failure in females, explained by protective blood-testis barrier. Both homozygous members presented positive GADAb but negative IA-2Ab and without T1D at present. The IA-2Abs seem to be the best predictors of T1D in APS-1, which were negative in our patients [33]; however, clinical evolution must be studied to confirm this data.

Although both siblings carried the same homozygous splicing mutation, the sister developed severe APS-1, whereas her brother presented mild symptoms. Previous reports have shown that different mutations in the *AIRE* gene lead to different phenotypes. For example, whereas most APS-1 Finnish patients present candidiasis, APS-1 Iranian Jews do not have this comorbidity and also do not show keratopathy [35]. Regarding these discrepancies, thirteen siblings from 6 families [36], [37] have currently been reported to show a different phenotypic spectrum of APS-1 carrying the same mutation, emphasizing that clinical features are variable not only among patients from different families but also among affected siblings. In the consanguineous Spanish family studied, both siblings have a homozygous mutation in the last nucleotide of the SSA of intron 5; this mutation raised the hypothesis that a different degradation between siblings of the mutated mRNA by the NMD could explain, in part, the different clinical outcomes of APS-1. On the contrary, the relative expression results showed a similar degradation of the mutated mRNA for both homozygous carriers, ruling out our hypothesis. Nonetheless, we consider that NMD must be contemplated when formulating and testing hypotheses concerning heterogeneous clinical outcomes, including situations of premature termination codon mutation, in order to provide appropriate genetic counseling regarding disease prognosis.

As for the case at hand, to understand the broad clinical phenotypes involved, even though the patients carried the same mutation, we must bear in mind that the ability of the immune system to recognize a huge number of antigenic determinants ensures a comprehensive protection against invading pathogens, but at the same time distinguishes between invading pathogens and self-antigens. Positive selection of T-lymphocytes with relatively low affinity for self-antigens in the thymus is a central event of this interplay, preventing errors that could lead to autoimmune diseases, such as T1D or APS-1. On the other hand, negative selection ensures that T-cells whose receptors recognize self-antigens with a high affinity are eliminated before they are exported to the periphery [38]. At this point, *AIRE* plays a crucial role, mediating negative selection of autoreactive T-cells in the thymus and also outside, as *AIRE* expression in the periphery provides a further path for the induction of T-cell tolerance [17].

Taking all this into account, the phenotypic diversity and disease severity of APS-1 patients carrying the same mutation in the same family could be due to the failure of negative selection that occurs at different moments between siblings and/or consequence of different environmental factors, such as viral infection on T-cell immune-tolerance education in peripheral tissues.

In conclusion, we report a new homozygous splicing mutation in the *AIRE* intron 5 acceptor (c.653-1G>A, in two patients of a consanguineous Spanish family with different phenotypes of APS-1. Our findings highlight the importance of sequencing splice sites surrounding the intron-exon junctions and the need to carry out additional RNA studies to ensure precise molecular diagnosis and correct genetic counseling. On the other hand, APS-1 diagnosis should be suspected and the *AIRE* gene studied in cases of childhood chronic candidiasis and/or hypoparathyroidism that is otherwise unexplained, especially when these clinical features are associated with other autoimmune diseases.

## Supporting Information

Table S1 **Nucleotide sequences of primers used for amplification and sequencing of** ***AIRE*** **exons and cDNA experiments.**
(DOCX)
