# Supplementary material for: New Splice Site Acceptor Mutation in AIRE Gene in Autoimmune Polyendocrine Syndrome Type 1
Source: PLoS One. 2014 Jul 2;9(7):e101616. doi: 10.1371/journal.pone.0101616 (PMC4079332; doi:10.1371/journal.pone.0101616)
Supplement: Table S1 — Nucleotide sequences of primers used for amplification and sequencing of AIRE exons and cDNA experiments. (DOCX) [file pone.0101616.s001.docx]

**Table S1.** Nucleotide sequences of primers used for amplification and sequencing of *AIRE* exons and cDNA experiments.

| **PRIMER** | **5' - 3' SEQUENCE** | **PRIMER** | **5' - 3' SEQUENCE** |
| --- | --- | --- | --- |
| hFw1-AIRE | CGGAGCGGCCTTTGCTCTT | hRv10-AIRE | GTAGGTCCTGGGCTCCTTGAA |
| hRv1-AIRE | GGGACTATCCCTGGCTCACAG | hFw11-AIRE | TCGGGTTGAGCTACATTTCC |
| hFw2-AIRE | GTTAGAAGTTGGTCCCCTTCC | hRv11-AIRE | GTGTGGTTGTGGGCTGTATG |
| hRv2-AIRE | GCTGAGCAGGTGACAGCA | hFw12-AIRE | GAGGTGGCACTCCTGCTC |
| hFw3-AIRE | ACCCTACCCCTGGAGAAAAC | hRv12-AIRE | TCTGCCCTGAGATGTGCTC |
| hRv3-AIRE | AGTGCTGGGTAGTCCCTTTG | hFw13-AIRE | ATCTCAGTGTGGGGGAAACA |
| hFw4-AIRE | CAAAGGGACTACCCAGCACT | hRv13-AIRE | CTGAGTTTCCACGGCTCAAG |
| hRv4-AIRE | CCCCTAGGACAGGGTCTCA | hFw14a-AIRE | GGAGGTTCTCACCGTCACTC |
| hFw5-AIRE | GCCTGCTTCTGGCATAGAGT | hRv14a-AIRE | AGTAGGTCACCAGGCAAGGA |
| hRv5-AIRE | GTGGTCCTCCTTCCATCTTG | hFw14b-AIRE | AATTAAACCCTGCCCCACTT |
| hFw6-AIRE | CCTGGGGCCTACACGACT | hRv14b-AIRE | TCCATTCAGGAAGCTGGAAC |
| hRv6-AIRE | CCAGAGGCAACGCTGTTC | qh-Fw4+5-AIRE | GAACGGGATTCAGACCATGT |
| hFw7-AIRE | CTCTGGGGGAGTGGCTCT | qh-Rv7-AIRE | GCCTAGCCTCACCTCCA |
| hRv7-AIRE | CCCTGAGTGCCCAGGTAAA | qh-Fw5+6-AIRE | GAGTCAGGCGGCTCCAA |
| hFw8-AIRE | AAGGAGGTGGCTCTCAGGA | qh-Rv6-AIRE | TGCCGGAGTCTTCGAACTT |
| hRv8-AIRE | TTCCATCTTGGATGGGAGAG | qh-Fwmut-AIRE | CATCCAGCAGGCAGGTC |
| hFw9-AIRE | CGCTGTCTTGTTCTGCATGT | qh-FwBact | CAGCCATGTACGTTGCTATCCAGG |
| hRv9-AIRE | ACAGGACTCCAGGGGACAG | qh-RvBact | AGGTCCAGACGCAGGATGGCATG |
| hFw10-AIRE | CACTGACTCCTGGGTGGTG |  |  |
